# Vegetable, fruit and antioxidant nutrient consumption and subsequent risk of hepatocellular carcinoma: a prospective cohort study in Japan

**DOI:** 10.1038/sj.bjc.6604843

**Published:** 2009-01-06

**Authors:** N Kurahashi, M Inoue, M Iwasaki, Y Tanaka, M Mizokami, S Tsugane

**Affiliations:** 1 Epidemiology and Prevention Division, Research Center for Cancer Prevention and Screening, National Cancer Center 5-1-1, Tsukiji Chuo-ku, Tokyo 104-0045, Japan; 2 Department of Clinical Molecular Informative Medicine, Nagoya City University Graduate School of Medical Sciences, Kawasumi, Mizuho, Nagoya 467-8601, Japan

**Keywords:** hepatocellular carcinoma, vegetables, fruits, carotenoid, vitamin C, prospective study.

## Abstract

In a population-based prospective study of 19 998 Japanese individuals, consumption of vegetables, green–yellow and green leafy vegetables was inversely associated with the risk of hepatocellular carcinoma (101 cases), with multivariable hazard ratios for the highest *vs* lowest tertile of 0.61 (95% confidence interval (CI)=0.36–1.03, *P*_trend_=0.07), 0.65 (95% CI=0.39–1.08, *P*_trend_=0.06) and 0.59 (95% CI=0.35–1.01, *P*_trend_=0.04), respectively.

Although the potential roles of fruits and vegetables in cancer prevention have been demonstrated at various cancer sites (Vainio and Weiderpass, 2006), the association with hepatocellular carcinoma (HCC) remains unclear (World Cancer Research Fund/American Institute for Cancer Research, 2007). Fruits and vegetables are a rich source of antioxidants, such as retinol and carotenoids, and vitamin C, and they are thought to exert protective effects against cancer (Stanner *et al*, 2004). In an intervention study, however, not all antioxidant nutrients might be protective against HCC (Bjelakovic *et al*, 2004).

Here, we investigated the association between fruit and vegetable consumption and HCC in a large-scale population-based cohort study in Japan, with due consideration for hepatitis C virus (HCV) and hepatitis B virus (HBV) infection status.

## Materials and methods

The Japan Public Health Center-based Prospective Study (JPHC study) Cohort II, initiated during 1993–1994, has been described earlier (Kurahashi *et al*, 2009). The study population was defined as all residents aged 40–69 years who lived in six PHC areas at the start of the baseline survey. We enrolled 56 635 men and women who provided valid responses to a self-administered questionnaire (82%) and excluded participants with a history of cancer (*n*=1219). Among them, a total of 20 406 participants (36%) provided a blood sample. These plasma samples were screened for anti-HCV and for HBV antigen (HBsAg).

The self-administered food-frequency questionnaire (FFQ) consisted of 52 foods, including beverages. It asked about the usual consumption of six vegetable and three fruit items during the previous year. The vegetables included two pickled vegetables (green leafy vegetables and other vegetables), green leafy vegetables (spinach, Chinese chives, etc), carrot, tomato and 100% vegetable juice, whereas the fruit items included apple, citrus fruits and 100% fruit juice. The questionnaire contained five frequency categories for vegetable and fruit consumption ranging from ‘never’ to ‘almost every day’, except for juices. Standard portion sizes were specified for each food item, which were then used to determine the three choice amounts of small (50% smaller), medium (same as the standard) and large (50% larger). Six frequency choices for juice ranged from ‘almost never’ to ‘5 or more cups per day’. The consumption of total fruit and total vegetables (g day^−1^) was calculated from these responses. We documented the validity of the FFQ in the assessment of vegetable and fruit consumption in subsamples using dietary records. Although validities for vegetables and fruits were relatively low (from 0.22 for vegetables to 0.31 for fruit), correlation coefficients for antioxidant nutrients were considered moderate (from 0.31 for vitamin C to 0.41 for *β*-carotene).

Among the 20 406 participants who responded to the questionnaire and provided a blood sample, 408 who reported extreme total energy intake (upper 1.0% or lower 1.0%) were excluded, leaving 19 998 participants for analysis, who were followed from the baseline survey until 31 December 2005. Of these, 5% moved out of a study area and 0.2% were lost to follow-up during the study period.

We used Cox regression to compute hazard ratios (HRs) and 95% confidence intervals (CIs) of HCC according to tertiles of consumption of the respective food items or nutrients with adjustment for potential confounders, including HCV or HBV infection status.

## Results

During 235 811 person-years of follow-up (11.8 years), a total of 101 new HCC cases were identified. The prevalence of chronic HCV and HBV infection in HCC cases was 70.3 and 12.9%, respectively.

We observed that participants with higher vegetable and fruit consumption tended to be older, smoke less, drink less alcohol, and consume less coffee and more genistein. Body mass index did not substantially differ according to consumption. The proportion of participants positive for anti-HCV, HBsAg or both among tertiles of vegetable and fruit consumption was similar. The prevalence of positive markers for HCV and HBV in this cohort was 5.3 and 2.5%, respectively.

Table 1 presents HRs in relation to vegetable and fruit consumption for HCC cases. Borderline inverse associations were seen between vegetables and green–yellow vegetables and HCC, with multivariable HRs for the highest *vs* lowest tertile of 0.61 (95% CI=0.36–1.03, *P*_trend_=0.07) and 0.65 (95% CI=0.39–1.08, *P*_trend_=0.06), respectively. In particular, green leafy vegetable consumption showed an inverse dose-dependent association with HCC (HR=0.59, 95% CI=0.35–1.01 for highest *vs* lowest tertile of consumption, *P*_trend_=0.04). Results for vegetables excluding pickled vegetables were similar to those for when they were included. In contrast, fruit consumption including fruit juice appeared to increase the risk of HCC, albeit without statistical significance (HR=1.45, 95% CI=0.85–2.48 for highest *vs* lowest tertile of consumption).

Table 2 shows the association between retinol, carotenoids (*α*-carotene and *β*-carotene) and vitamin C and HCC risk. A slightly negative association was seen between *α*- and *β*-carotene and HCC, with respective multivariable HRs for the highest *vs* lowest tertile of 0.69 (95% CI=0.42–1.15) and 0.64 (95% CI=0.38–1.08). Multivariable HR for vitamin C was somewhat increased in the highest category (HR=1.38, 95% CI=0.80–2.40).

When the analysis was restricted to participants who were either or both anti-HCV- or HBsAg-positive, these results were substantially unchanged. It is worth noting that our study showed that the preventive effects of *α*- and *β*-carotene on HCC strengthened, with respective multivariable HRs for the highest *vs* lowest tertile of 0.60 (95% CI=0.34–1.08, *P*_trend_=0.08) and 0.61 (95% CI=0.34–1.09, *P*_trend_=0.08) (data not shown).

After participants were stratified by smoking status, multivariable HRs for the highest *vs* lowest tertile among never smokers were 0.42 for vegetables (95% CI=0.19–0.99, *P*_trend_=0.03), 0.30 for green–yellow vegetables (95% CI=0.13–0.70, *P*_trend_<0.01) and 0.31 for green leafy vegetables (95% CI=0.13–0.74, *P*_trend_<0.01). Regarding nutrients, *β*-carotene showed a significant inverse association with risk among never smokers (highest *vs* lowest: HR=0.31, 95% CI=0.13–0.76). In contrast, vitamin C seemed to be positively associated with HCC risk among current smokers, with an increase in multivariable HR for HCC in the second and highest categories (HR=3.58, 95% CI=1.21–10.63 and HR=2.69, 95% CI=0.89–8.08, respectively) (data not shown).

## Discussion

Our study identified inverse associations between the consumption of vegetables, green–yellow and green leafy vegetables and HCC. Concomitantly, an inverse association between *α*- and *β*-carotene and HCC risk was shown. These results are plausible, given the abundance of these nutrients in vegetables, particularly green–yellow vegetables.

In an animal experiment, carotenoids were shown to suppress liver carcinogenesis (Murakoshi *et al*, 1992; Moreno *et al*, 2002), whereas in an intervention study in patients with viral hepatitis and cirrhosis, a greater than 50% decrease in HCC incidence was found in the group administered a carotenoid mixture in addition to conventional treatment compared with a group given conventional symptomatic treatment alone (placebo not used) (Nishino, 2007). These findings support our present findings. It is worth noting that our study showed that the preventive effects of *α*- and *β*-carotene on HCC were strengthened when participants were limited to those who were either or both HBV and HCV positive. Given that inflammation is accompanied by the excess production of free radicals and that carotenoids have antioxidant potential in the scavenging of free radicals (Krinsky, 1989), carotenoids appear to play an important role in the prevention of hepatitis virus infection-related liver carcinogenesis.

In contrast, vitamin C consumption appeared to be associated with an increased risk of HCC. These relations were strengthened among current smokers in our study (see Results). Although vitamin C has antioxidant potential, it also acts to stimulate the absorption of iron from food (Lynch, 1997), and iron overload is considered a risk factor for HCC (Kowdley, 2004). Dietary vitamin C is positively associated with ferritin, which was used as a measure of body iron stores in the study by Fleming *et al* (1998). Thus, a higher intake of vitamin C might be harmful to hepatic cells, especially among smokers.

Given that the prognosis for HCC is extremely poor, our results would, if confirmed, have important implications for public health. Greater consumption of vegetables that contain *α*- and *β*-carotene and restraint in those rich in vitamin C may modify the development of HCC in HBV- and/or HCV-infected participants.

## Figures and Tables

**Table 1:** Hazard ratio and 95% confidence intervals for hepatocellular carcinoma according to tertile of intake of vegetables and fruits, JPHC study (*n*=19 998)

	**Lowest**	**Middle**	**Highest**	***P*_trend_**
*Total vegetables and fruits*				
Median (g day^−1^)	55.3	120.3	200.9	
No. of cases/person-years of follow-up	32/79 057	22/78 938	47/77 816	
Age, area, sex-adjusted HR (95% CI)	1.00	0.71 (0.41–1.23)	1.23 (0.78–1.94)	0.38
Multivariate HR^a^ (95% CI)	1.00	0.78 (0.45–1.38)	1.14 (0.70–1.86)	0.56
				
*Vegetables*				
Median (g day^−1^)	25.6	51.7	88.5	
No. of cases/person-years of follow-up	37/78 971	31/79 183	33/77 657	
Age, area, sex-adjusted HR (95% CI)	1.00	0.88 (0.55–1.43)	0.81 (0.50–1.29)	0.37
Multivariate HR^b^ (95% CI)	1.00	0.79 (0.48–1.31)	0.61 (0.36–1.03)	0.07
				
*Green–yellow vegetables*				
Median (g day^−1^)	10.1	23.1	42.3	
No. of cases/person-years of follow-up	44/78 234	24/79 272	33/78 305	
Age, area, sex-adjusted HR (95% CI)	1.00	0.66 (0.40–1.09)	0.81 (0.51–1.28)	0.27
Multivariate HR^b^ (95% CI)	1.00	0.55 (0.33–0.94)	0.65 (0.39–1.08)	0.06
				
*Green leafy vegetables*				
Median (g day^−1^)	7.1	17.0	32.3	
No. of cases/person-years of follow-up	42/78 473	31/79 018	28/78 320	
Age, area, sex-adjusted HR (95% CI)	1.00	0.82 (0.51–1.30)	0.72 (0.44–1.17)	0.17
Multivariate HR^b^ (95% CI)	1.00	0.71 (0.44–1.17)	0.59 (0.35–1.01)	0.04
				
*Fruit*				
Median (g day^−1^)	13.4	68.0	120.3	
No. of cases/person-years of follow-up	29/78 795	25/78 872	47/78 144	
Age, area, sex-adjusted HR (95% CI)	1.00	0.91 (0.53–1.56)	1.30 (0.81–2.09)	0.32
Multivariate HR^c^ (95% CI)	1.00	1.08 (0.61–1.91)	1.45 (0.85–2.48)	0.19
				
*Fruit excluding 100% fruit juice*				
Median (g day^−1^)	11.8	46.8	97.2	
No. of cases/person-years of follow-up	32/78 489	26/78 961	43/78 361	
Age, area, sex-adjusted HR (95% CI)	1.00	0.97 (0.58–1.65)	1.24 (0.77–1.99)	0.40
Multivariate HR^c^ (95% CI)	1.00	0.79 (0.45–1.38)	1.08 (0.65–1.82)	0.81

CI=confidence interval; HBsAg=HBV antigen; HCV, hepatitis C virus; HR=hazard ratio.

a Adjusted for age, area, sex, HCV, HBsAg, smoking status, alcohol consumption, body mass index, history of diabetes mellitus and intake of coffee, genistein.

b Adjusted for age, area, sex, HCV, HBsAg, smoking status, alcohol consumption, body mass index, history of diabetes mellitus and intake of coffee, genistein and fruit.

c Adjusted for age, area, sex, HCV, HBsAg, smoking status, alcohol consumption, body mass index, past history of diabetes mellitus and intake of coffee, genistein and vegetable.

**Table 2:** Hazard ratio and 95% confidence intervals for hepatocellular carcinoma according to tertile of intake of nutrient, JPHC study (*n*=19 998)

	**Lowest**	**Middle**	**Highest**	***P*_trend_**
*Retinol*				
Median (mg day^−1^)	114.8	282.7	397.2	
No. of cases/person-years of follow-up	33/78 650	34/78 824	34/78 338	
Age, area, sex-adjusted HR (95% CI)	1.00	1.24 (0.75–2.03)	1.37 (0.84–2.23)	0.20
Multivariate HR^a^ (95% CI)	1.00	1.26 (0.76–2.10)	1.07 (0.64–1.79)	0.65
				
α-*carotene*				
Median (mg day^−1^)	50.4	146.6	561.2	
No. of cases/person-years of follow-up	40/78 660	28/78 756	33/78 395	
Age, area, sex-adjusted RR (95% CI)	1.00	0.78 (0.48–1.27)	0.81 (0.51–1.29)	0.34
Multivariate HR^a^ (95% CI)	1.00	0.73 (0.44–1.22)	0.69 (0.42–1.15)	0.14
				
β-*carotene*				
Median (mg day^−1^)	602.2	1355.7	2319.0	
No. of cases/person-years of follow-up	39/78 628	30/79 082	32/78 101	
Age, area, sex-adjusted HR (95% CI)	1.00	0.87 (0.54–1.41)	0.79 (0.49–1.26)	0.31
Multivariate HR^a^ (95% CI)	1.00	0.82 (0.50–1.35)	0.64 (0.38–1.08)	0.10
				
*Vitamin C*				
Median (mg day^−1^)	36.4	67.8	93.9	
No. of cases/person-years of follow-up	23/78 495	34/78 964	44/78 352	
Age, area, sex-adjusted HR (95% CI)	1.00	1.41 (0.82–2.40)	1.33 (0.79–2.24)	0.39
Multivariate HR^a^ (95% CI)	1.00	1.74 (0.996–3.06)	1.38 (0.80–2.40)	0.44

CI=confidence interval; HBsAg=HBV antigen; HCV, hepatitis C virus; HR=hazard ratio.

a Adjusted for age, area, sex, HCV, HBsAg, smoking status, alcohol consumption, body mass index, history of diabetes mellitus and intake of coffee and genistein.
